# Pathological Features of Localized Prostate Cancer in China: A Contemporary Analysis of Radical Prostatectomy Specimens

**DOI:** 10.1371/journal.pone.0121076

**Published:** 2015-03-23

**Authors:** Yao Zhu, Xiao-Qun Yang, Cheng-Tao Han, Bo Dai, Hai-Liang Zhang, Guo-Hai Shi, Chao-Fu Wang, Ding-Wei Ye

**Affiliations:** 1 Department of Urology, Fudan University Shanghai Cancer Center, Shanghai, 200032, China; 2 Department of Oncology, Shanghai Medical College, Fudan University, Shanghai, 200032, China; 3 Department of Pathology, Fudan University Shanghai Cancer Centre, Shanghai 200032, China; Istituto dei tumori Fondazione Pascale, ITALY

## Abstract

There has been a rapid increase in the incidence of prostate cancer in China, especially in areas with boosted economic development. In this study, we analyzed the pathological features of a contemporary series of radical prostatectomy cases. A total of 230 consecutive, whole-mounted radical prostatectomy specimens collected from 2012 to 2014 were reviewed. The median age of the patients was 68 years, and 64.3% of patients presented with prostate specific antigen alone. Pathological examination indicated that a high proportion (77.4%) of patients had intermediate- or high-risk disease according to the Cancer of the Prostate Risk Assessment Post-Surgical score. After surgery, only 28 patients met the criteria for active surveillance (organ-confined Gleason ≥6 disease). The Prostate Cancer Research International Active Surveillance criteria achieved a sensitivity of 57.1% and a specificity of 98.0% for identifying candidates. The probability of Gleason score upgrading was 24.8% in the entire group and 59.0% in biopsy-confirmed Gleason ≥6 disease. The predominant tumor was located in the transition zone in 14.8% of cases, while only three patients (1.3%) had a predominant tumor located in the anterior region. Patients with transition zone-predominant tumor were likely to have been referred with urinary symptoms and high prostate specific antigen levels. The results of this study highlight the contemporary pathological features of localized prostate cancer in urban China. There was an increased trend towards asymptomatic cases, though most patients had intermediate- or high-risk disease and were suitable for definitive treatment. The low prevalence of dominant cancer in the anterior region may reflect race-based pathological differences.

## Introduction

Prostate cancer (PCa) was regarded as a rare disease in China as recently as a decade ago, with a reported incidence of 1.6 per 10,000 [1]. However, the rate increased to 5.3 per 10,000 in 2012, with 47,000 new cases annually [2]. The most rapid increase in PCa incidence was observed in areas with boosted economic development [3]. For instance, the Shanghai cancer registry reported an annual increase of 8% in PCa incidence over the last decade, with PCa ranked as the fifth most common cancer in men. A better understanding of the disease characteristics of PCa is therefore essential to allow us to face this emerging challenge.

Currently, there is limited information available regarding the characteristics of PCa in China, making it difficult to compare the emerging threat with that in Western countries. In 2005, a multicenter study examined PCa cases, most of which were diagnosed in Shanghai [4]. They found that nearly 90% of Chinese PCa patients presented with urinary symptoms or bone pain. The median prostate specific antigen (PSA) level was 46 ng/ml and at least 36% of patients had metastases at diagnoses. Of 431 patients, 336 (78%) underwent androgen deprivation therapy and only 24 (6%) received radical prostatectomy (RP). The high percentage of advanced disease reinforced the emphasis on systematic treatment. However, new data have revealed a dramatic shift in cancer stage and management. The cancer registry in urban Shanghai from 2000 to 2009 showed that 86% of patients had nonmetastatic disease at diagnosis, and RP was performed in 43% of subjects [5]. These changes in disease pattern indicate the need for further research on localized disease.

In this study, we evaluated a recent consecutive RP cohort with detailed pathological data. We considered the following issues in the context of Chinese subjects: the current pathological characteristics and risk profile of localized disease; the feasibility and performance of active surveillance criteria; and tumor distribution and its possible implications for diagnosis and treatment.

## Materials and Methods

Since the whole-mount technique was established in our institution in 2012, prostatectomy specimens have been prospectively examined and detailed clinicopathological information has been maintained in an electronic database. Information on patients treated from December 2012 to July 2014 was retrieved to analyze the contemporary features of localized PCa. Cases diagnosed as nonadenocarcinoma or treated with neoadjuvant therapies and prior prostate surgery were excluded from the study. Slides were reviewed by two experienced pathologists (W.C.F and Y.X.Q) (trained in pathology at Massachusetts General Hospital), who were blinded to the clinical and biopsy information. As a tertiary cancer center, we treat a high volume of RP cases in mainland China (1,257 patients as of July 2014). The study was approved by the institution’s ethics committee and all patients provided written informed consent.

RP was performed by four board-certified urologists with at least 10 years of experience. In our center, RP was indicated in men with clinically-localized disease and good physical fitness. Higher chronological age alone was not an absolute contraindication for RP. Each prostate specimen was serially sectioned into 2–3-mm-thick axial slices, embedded in paraffin, and a single 5-μm slice was cut from each thick section using a microtome. The thin slices were then attached to glass slides and processed by staining with hematoxylin and eosin. After pathological review, the following information was evaluated: tumor location (lobe, apex, transition zone [TZ], and anterior region [AR]), Gleason score (primary, secondary and tertiary grades), pathological stage, tumor volume, perineural invasion, lymphovascular invasion, surgical margin (apex and base), and lymph node metastases. A four-category classification was used to describe tumor distribution in specific regions of the prostate: no tumor, <25% of tumor, 25–75% of tumor, and >75% of tumor. Only cases with >75% of tumor located in the TZ and AR were classified as TZ- and AR-predominant (TZp and ARp), respectively.

Disease profile was characterized using the Cancer of the Prostate Risk Assessment Post-Surgical score (CAPRA-S) [6], which is strongly associated with biochemical progression risk after RP. The comprehensive score was the sum of six variables: PSA level, surgical margin (SM), seminal vesicle invasion, Gleason score, extracapsular extension, and lymph node invasion. Quantitative risk score was grouped for clinical practice: 0–2 indicated relatively low-risk, 3–5 intermediate-risk, and ≥6 high-risk disease. Based on a previous report in Asian patients [7], the Prostate Cancer Research International Active Surveillance (PRIAS) criteria were used to select cases suitable for active surveillance. Baseline tumor characteristics should meet the following requirements: PSA ≤10.0 ng/ml, PSA density <0.2 ng/ml, stage T1c/T2, Gleason score ≤3+3 = 6, and ≤2 positive biopsy cores.

Statistical analyses were performed using R software. χ^2^ tests were used to examine the relationships between categorical variables, and *t*-tests were used for continuous variables. PSA was log-transformed to ensure a normal distribution.

## Results

### Pathological characteristics and risk profile of localized PCa

During the 20-month study period, a total of 230 PCa patients fulfilled the inclusion criteria for further analyses. The baseline clinicopathological characteristics are shown in Table 1. Half of the patients (50.4%) were in the 60–70-year age range, whereas only 16.5% were younger than 60 years. Before surgery, 29.1% of patients had PSA levels >20 ng/ml. The diagnosis of PCa was suspected based on increased PSA without symptoms in 64.3% of patients. Clinicopathological features were compared between symptomatic patients and patients with elevated PSA alone (S1 Table). Patients referred with PSA alone were slightly younger but had comparable PSA levels to symptomatic patients. There were no significant differences in tumor stage and Gleason score between the two presenting patterns.

**Table 1:** Patient demographics.

Variables	Statistics
No.	230
Age (median, range)	68, 41–81
Presenting symptom (n, %)	
PSA alone	148, 64.3%
Urinary	66, 28.7%
Other	16, 7.0%
Family history of PCa (n, %)	4, 1.7%
Serum PSA (median, range)	12.7, 0.5–154.7
BMI (median, range)	23.5, 16.9–32.4
pT stage (n, %)	
T2a	41, 17.8%
T2b	6, 2.6%
T2c	93, 40.4%
T3a	45, 19.6%
T3b	45, 19.6%
pN1 (n, %)	14, 6.1%
Gleason score (n, %)	
3+3	29, 12.6%
3+4	85, 37.0%
4+3	62, 27.0%
8–10	54, 23.5%

Abbreviations: PSA, prostate specific antigen; PCa, prostate cancer; BMI, body mass index.

Pathological examination of RP specimens showed that 39.1% of patients had stage T3 disease and 23.5% had a Gleason score ≥8. According to CAPRA-S, 52 (22.6%) patients had low-risk disease, 92 (40%) were intermediate-risk and 86 (37.4%) were high-risk. Positive SM was found in 64 patients and influenced the CAPRA-S risk attributions in 25, which constituted 10.9% of total cases. Because of positive SMs, 7.1% of low-risk and 19.4% of intermediate-risk patients were upgraded to a higher classification.

### Feasibility and performance of active surveillance criteria

Pathological examination of RP specimens revealed only 28 (12.2%) patients had organ-confined (T2) Gleason ≤6 disease. The performance of PRIAS criteria for identifying candidates fit for active surveillance was examined, and yielded a sensitivity of 57.1%, a specificity of 98.0%, a positive predictive value of 80%, and a negative predictive value of 94.3%. In 20 (8.7%) patients who fulfilled the PRIAS criteria before surgery, four had unfavorable disease features in postoperative reports. Although no increase in T stage was found, the Gleason score was upgraded to 7 in these four patients.

Biopsy and RP specimen Gleason scores are shown in Table 2. Gleason score upgrade occurred in 24.8% of patients. Importantly, the probability of upgrade was significantly higher in patients with a biopsy Gleason score of ≤6 (p<0.01). More than half had adverse grades in RP specimens.

**Table 2:** Paired comparison of biopsy and radical prostatectomy Gleason scores in Chinese patients with localized prostate cancer (n = 230). *

Biopsy Gleason score	RP Gleason score				Total number
	≤6	3+4	4+3	≥8	
≤6	25, 41.0%	**27, 44.3%**	**7, 11.5%**	**2, 3.3%**	61
3+4	2, 3.6%	39, 70.9%	**10, 18.2%**	**4, 7.3%**	55
4+3	1, 2.4%	13, 31.0%	21, 50%	**7, 16.7%**	42
≥8	1, 1.4%	6, 8.3%	24, 33.3%	41, 56.9%	72
Total number	29	85	62	54	230

*percentage was calculated according to row.

Cases had Gleason score upgrading were indicated in bold formatting.

Abbreviations: RP, radical prostatectomy.

### Tumor distribution and possible predictors

The predominant tumor was located in the TZ in 14.8% (34) of cases. TZp was strongly associated with urinary symptoms at presentation and increased PSA levels (Table 3). There were no strong associations between TZ location and other prediagnostic characteristics including age, obesity, smoking history, alcohol consumption, hypertension and diabetes mellitus (Table 3). Notably, TZp tumors showed a high likelihood of high grade and adverse features characterized by CAPRA-S score.

**Table 3:** Association between transition zone-predominant tumor and clinicopathological features.

Variables	Transition zone predominate		p-value
	Yes (n = 34)	No (n = 196)	
Urinary symptom	15	51	0.03
PSA level, mean	28.22	12.06	<0.01
Age, mean	68.94	65.33	0.06
BMI, mean	23.37	23.56	0.70
Smoking history	12	59	0.55
Alcohol consumption	4	34	0.42
Hypertension	15	81	0.76
Diabetes mellitus	5	14	0.14
RP Gleason score			0.02
6	0	29	
7	22	125	
8–10	12	42	
CAPRA-S			<0.01
low-risk	1	51	
intermediate-risk	11	81	
high-risk	22	64	

Abbreviations: PSA, prostate specific antigen; BMI, body mass index; RP, radical prostatectomy; CAPRA-S, Cancer of the Prostate Risk Assessment Post-Surgical Score.

In this series, only three (1.3%) patients had a predominant tumor located in the AR. Two had Gleason 7 disease and one had Gleason 6 disease. According to CAPRA-S classification, one had low-risk disease and two had intermediate-risk disease.

Table 4 summarizes a recent report on the tumor distributions in RP specimens across different populations. Chinese patients demonstrated a similar probability of TZp to other populations, but a relatively lower risk of ARp PCa (Table 4).

**Table 4:** Tumor distributions in radical prostatectomy specimens among different populations.

***Transition zone predominant***					
Reference	Year of publication	Country	Racial groups	Total number	Percentage
[8]	2002	United States	n.m.	317	9.8%
[9]	2005	United States	n.m.	62	16.1%
[10]	2009	United States	n.m.	494	18.0%
[11]	2006	Japan	Japanese	185	14.6%
[12]	2012	Japan	Japanese	92	32.6%
[13]	2014	Japan	Japanese	211	35.3%
Current study	2014	China	Chinese	230	14.8%
***Anterior region predominant***					
[9]	2005	United States	n.m.	62	16.1%
[14]	2008	United States	n.m.	1312	15.0%
[15]	2014	United States	White	1066	10.6%
			Black	403	9.7%
[16]	2014	United States	White	89	28.7%
			Black	87	50.6%
Current study	2014	China	Chinese	230	1.3%

Abbreviations: n.m., not mentioned.

## Discussion

Despite the emerging threat of PCa in developed areas of China, detailed information on its characteristics and potential challenges remain scarce. In the current study, we performed detailed pathological evaluations in a contemporary cohort of Chinese PCa patients who underwent RP, to provide an accurate definition of disease characteristics. These findings not only provide up to date information regarding localized PCa in China, but will also help to decide on treatment emphasis.

This study showed that a significant proportion of Chinese PCa patients were diagnosed with increasing PSA, without symptoms. This situation differs from that seen a decade ago, when Peyromaure et al. reported that only 6.2% of Chinese patients had asymptomatic PCa [4]. This dramatic shift may be explained by the increased use of PSA tests in metropolitan areas, associated with the steadily increasing incidence of PCa. For example, the adjusted incidence rate of PCa in Shanghai was 13/100,000 men in 2009, compared with 5.6/100,000 in 1999 [3]. PCa is now ranked as the most common genitourinary malignancy, and PSA tests are accordingly prescribed more frequently in clinical practice. However, it should be noted that most cases of localized PCa in China had high PSA and poor risk profiles; for example, 37.4% of our patients had CAPRA-S high-risk disease with predicted 5-year progression-free survival of <25% [6]. A recent national database from the United States however, showed similar risk attributions in only 6.5% of cases [6]. The reasons for this difference are multifactorial. First, PSA screening is widely acknowledged by the public and annual testing often starts at age 50 years in Western countries. In contrast, PCa awareness in Chinese society remains low, and patients usually only receive PSA testing as an additional examination, rather than as routine screening. Second, several reports have shown that a PSA cut-off value of 4 ng/ml resulted in a high false-positive rate in Chinese men [17]. Most studies were conducted in tertiary centers with a high incidence of prostatitis, and a trigger cut-off of 10 ng/ml for prostate biopsy was therefore recommended by Chinese guidelines and is often used in community settings. Some patients may therefore not undergo prostate biopsy until their PSA is ≥10 ng/ml.

This study provided the first assessment of the feasibility of active surveillance criteria in Chinese patients. As expected, active surveillance could rarely be applied in unscreened cohorts. Only 12.2% of patients had organ-confined Gleason ≤6 disease. However, PRIAS criteria showed promising results for identifying Chinese PCa cases with favorable tumor features. If this high specificity could be replicated in large, multicenter series, the PRIAS criteria could be used in clinical practice for selecting candidates for expectant management. We also evaluated the probability of Gleason score upgrade, which may be helpful for planning definitive treatment. Our results showed a high risk of upgrading in biopsy-favorable disease in Chinese patients; specifically, 57.4% of biopsy-confirmed Gleason 3+3 disease cases were upgraded after RP, compared with 36.3% in the United States [18]. Less-intensive treatment such as dose reduction in radiotherapy and omitting lymphadenectomy during RP, should thus be carefully discussed in biopsy-low-risk Chinese patients.

PCa tends to be multifocal, with a predilection for the peripheral zone. Tumors predominant to the TZ or AR, however, are less likely to be detected by standard biopsy procedures and may be associated with a higher incidence of adverse pathological features [11,15]. TZp tumors were reported in 20% of PCa patients from Western countries, while a contemporary series from Japan showed a remarkably high probability of TZp disease [13]. The current Chinese series found a similar prevalence of TZp tumors to that found in Western series. We evaluated the preoperative characteristics associated with TZp tumors and observed an increased probability of urinary symptoms and high PSA levels. The high PSA levels and adverse pathological findings in RP specimens may have been caused by a delay in diagnosis using conventional procedures. These results therefore suggest that additional TZ sampling may be advisable in patients with increased risk indicated by persistent urinary symptoms or increasing PSA after negative prior biopsy. We found a low probability of ARp tumors in this Chinese population. Sundi et al. previously reported a remarkable racial difference in the prevalence of ARp tumors [16], with Black men more likely to have ARp than Caucasians (51% vs. 29%, p = 0.003). Our results extend these earlier findings and suggest the need for further studies to validate these observations.

The major limitation of the current study was the potential selection bias associated with the single-center design and short time span. However, as a regional PCa center, we treat a large number of patients and employ well-trained surgeons and pathologists. Furthermore, qualified processing techniques for RP specimens are rarely available outside high-volume centers in China.

In conclusion, the results of this study provide information on the contemporary pathological features of localized PCa in urban China. An increased trend towards asymptomatic cases was observed, though most still presented with intermediate- and high-risk disease. Active surveillance is impractical in most Chinese patients, and Gleason upgrade is frequently observed in tumors with biopsy score ≤6 disease. The probability of TZp tumors was similar in Chinese and Western patients, while ARp tumors were rare.

## Supporting Information

S1 Table Comparison of clinicopathological features according to presenting pattern in Chinese patients with localized prostate cancer. (DOCX)

## References

[1] Parkin DM, Bray F, Ferlay J, Pisani P. Global cancer statistics, 2002. CA Cancer J Clin. 2005;55: 74–108. 15761078 10.3322/canjclin.55.2.74

[2] Ferlay J, Soerjomataram I, Ervik M, Dikshit R, Eser S, Mathers C, et al GLOBOCAN 2012 v1.0, Cancer Incidence and Mortality Worldwide: IARC CancerBase No. 11; 2013 Lyon: France: International Agency for Research on Cancer [Internet] Available: http://globocan.iarc.fr.

[3] Zhu Y, Wang HK, Qu YY, Ye DW. Prostate cancer in East Asia: evolving trend over the last decade. Asian J Androl. 2015;17: 48–57. 10.4103/1008-682X.132780 25080928 PMC4291877

[4] Peyromaure M, Debre B, Mao K, Zhang G, Wang Y, Sun Z, et al Management of prostate cancer in China: a multicenter report of 6 institutions. J Urol. 2005;174: 1794–1797. 16217289 10.1097/01.ju.0000176817.46279.93

[5] Hu Y, Zhao Q, Rao J, Deng H, Yuan H, Xu B. Longitudinal trends in prostate cancer incidence, mortality, and survival of patients from two Shanghai city districts: a retrospective population-based cohort study, 2000–2009. BMC Public Health. 2014;14: 356 10.1186/1471-2458-14-356 24731197 PMC4005468

[6] Cooperberg MR, Hilton JF, Carroll PR. The CAPRA-S score: A straightforward tool for improved prediction of outcomes after radical prostatectomy. Cancer. 2011;117: 5039–5046. 10.1002/cncr.26169 21647869 PMC3170662

[7] Lee DH, Jung HB, Lee SH, Rha KH, Choi YD, Hong SJ, et al Comparison of pathological outcomes of active surveillance candidates who underwent radical prostatectomy using contemporary protocols at a high-volume Korean center. Jpn J Clin Oncol. 2012;42: 1079–1085. 10.1093/jjco/hys147 22988037

[8] Jack GS, Cookson MS, Coffey CS, Vader V, Roberts RL, Chang SS, et al Pathological parameters of radical prostatectomy for clinical stages T1c versus T2 prostate adenocarcinoma: decreased pathological stage and increased detection of transition zone tumors. J Urol. 2002;168: 519–524. 12131301

[9] Cheng L, Jones TD, Pan CX, Barbarin A, Eble JN, Koch MO. Anatomic distribution and pathologic characterization of small-volume prostate cancer (<0.5 ml) in whole-mount prostatectomy specimens. Mod Pathol. 2005;18: 1022–1026. 15861213 10.1038/modpathol.3800431

[10] King CR, Ferrari M, Brooks JD. Prognostic significance of prostate cancer originating from the transition zone. Urol Oncol. 2009;27: 592–597. 10.1016/j.urolonc.2008.05.009 18799332 PMC9034732

[11] Sakai I, Harada K, Kurahashi T, Yamanaka K, Hara I, Miyake H. Analysis of differences in clinicopathological features between prostate cancers located in the transition and peripheral zones. Int J Urol. 2006;13: 368–372. 16734852 10.1111/j.1442-2042.2006.01307.x

[12] Kimura T, Furusato B, Miki J, Yamamoto T, Hayashi N, Takahashi H, et al Expression of ERG oncoprotein is associated with a less aggressive tumor phenotype in Japanese prostate cancer patients. Pathol Int. 2012;62: 742–748. 10.1111/pin.12006 23121605

[13] Takahashi H, Epstein JI, Wakui S, Yamamoto T, Furusato B, Zhang M. Differences in prostate cancer grade, stage, and location in radical prostatectomy specimens from United States and Japan. Prostate. 2014;74: 321–325. 10.1002/pros.22754 24259155

[14] Al-Ahmadie HA, Tickoo SK, Olgac S, Gopalan A, Scardino PT, Reuter VE, et al Anterior-predominant prostatic tumors: zone of origin and pathologic outcomes at radical prostatectomy. Am J Surg Pathol. 2008;32: 229–235. 10.1097/PAS.0b013e31812f7b27 18223325

[15] Mygatt J, Sesterhenn I, Rosner I, Chen Y, Cullen J, Morris-Gore T, et al Anterior tumors of the prostate: clinicopathological features and outcomes. Prostate Cancer Prostatic Dis. 2014;17: 75–80. 10.1038/pcan.2013.54 24296774

[16] Sundi D, Kryvenko ON, Carter HB, Ross AE, Epstein JI, Schaeffer EM. Pathological examination of radical prostatectomy specimens in men with very low risk disease at biopsy reveals distinct zonal distribution of cancer in black American men. J Urol. 2014;191: 60–67. 10.1016/j.juro.2013.06.021 23770146 PMC4042393

[17] Na R, Wu Y, Xu J, Jiang H, Ding Q. Age-specific prostate specific antigen cutoffs for guiding biopsy decision in Chinese population. PLoS One. 2013;8: e67585 10.1371/journal.pone.0067585 23825670 PMC3692456

[18] Epstein JI, Feng Z, Trock BJ, Pierorazio PM. Upgrading and downgrading of prostate cancer from biopsy to radical prostatectomy: incidence and predictive factors using the modified Gleason grading system and factoring in tertiary grades. Eur Urol. 2012;61: 1019–1024. 10.1016/j.eururo.2012.01.050 22336380 PMC4659370

