# Supplementary material for: Pathological Features of Localized Prostate Cancer in China: A Contemporary Analysis of Radical Prostatectomy Specimens
Source: PLoS One. 2015 Mar 23;10(3):e0121076. doi: 10.1371/journal.pone.0121076 (PMC4370496; doi:10.1371/journal.pone.0121076)
Supplement: S1 Table — (DOCX) [file pone.0121076.s001.docx]

**Supplementary Table 1.** **Comparison of clinicopathological features according to presenting pattern in Chinese patients with localized prostate cancer.**

| Variables | Presenting pattern | | p-value |
| --- | --- | --- | --- |
|  | PSA alone (n=148) | Symptomatic (n=82) |  |
| PSA, mean | 13.87 | 13.74 | 0.94 |
| Age, mean | 65.39 | 67.06 | 0.24 |
| Body mass index, mean | 23.53 | 23.52 | 0.98 |
| Family history | 2 | 2 | 0.55 |
| pT stage |  |  | 0.71 |
| T2a | 27 | 14 |  |
| T2b | 5 | 1 |  |
| T2c | 62 | 31 |  |
| T3a | 26 | 19 |  |
| T3b | 28 | 17 |  |
| pN1 | 7 | 7 | 0.25 |
| RP Gleason score |  |  | 0.60 |
| 6 | 18 | 11 |  |
| 7 | 98 | 49 |  |
| 8-10 | 32 | 22 |  |

Abbreviations: PSA, prostate specific antigen; RP, radical prostatectomy.
